# Validation of serum cystatin SN detection for diagnosis and poor prognosis of esophageal squamous cell carcinoma

**DOI:** 10.3389/fonc.2024.1337707

**Published:** 2024-02-13

**Authors:** Yingqi Pi, Sizhuo Lin, Xiuqin Ren, Lin Wang, Yiling Song, Zhikun Wu, Yanzhen Lai

**Affiliations:** ^1^ Department of Clinical Laboratory, Sun Yat-sen University Cancer Center, Guangzhou, China; ^2^ Department of Clinical Laboratory, The Seventh Affiliated Hospital, Sun Yat-sen University, Shenzhen, China; ^3^ Department of Oncology, Heyuan People’s Hospital, Heyuan, China

**Keywords:** marker, cystatin-SN, ESCC, diagnosis, therapeutic effect, prognosis predictor

## Abstract

**Background:**

The identification of effective tumor markers is of paramount importance for the early diagnosis, treatment, and prognosis of esophageal squamous cell carcinoma (ESCC). The present study endeavors to identify efficacious serological markers that can differentiate patients with early-stage ESCC from those with benign esophageal lesions and healthy controls (HC). Cystatin-SN (CST1), an active cysteine protease inhibitor belonging to the Cystatin (CST) superfamily, is implicated in the pathogenesis of inflammation and tumorigenesis. The objective of this investigation is to assess the diagnostic, therapeutic, and prognostic potential of serum CST1 in ESCC.

**Methods:**

In our prior RNA sequencing and screening endeavors, we have identified ten genes that are up-regulated in relation to esophageal cancer. Subsequently, we have verified the gene CST1 from the transcriptome data of the The Cancer Genome Atlas Program (TCGA) and Gene Expression Profiling Interactive Analysis (GEPIA) database. Following this, we conducted an enzyme-linked immunosorbent assay (ELISA) to ascertain the expression levels of CST1 in serum samples from clinical cohorts.

**Results:**

The study revealed a significant elevation in serum CST1 levels among patients with early-stage esophageal squamous cell carcinoma (ESCC) (7.41 ± 4.32 ng/ml) compared to those with esophageal benign lesions (4.67 ± 2.43 ng/ml) (p < 0.0001) and healthy controls (4.87 ± 2.77 ng/ml) (p < 0.0001). The diagnostic sensitivity of CST1 for ESCC was 75.68% (specificity 70.83%, AUC 0.775). Combination of CST1 and SCC-Ag exhibited the AUC up to 0.819. Additionally, serum CST1 levels exhibited a significant decrease at 1-2 weeks post-surgery (4.49 ± 3.31 ng/ml) compared to pre-surgery levels (7.68 ± 3.71 ng/ml) (p<0.0001). Survival analysis demonstrated a strong association between high (844/415-1543 d) or low (1490/645-1710 d) serum CST1 levels at diagnosis and overall survival time (p < 0.001). Furthermore, multivariate regression analysis confirmed CST1 (p=0.024, HR=2.023, 95%CI 1.099–3.725) as an independent prognostic factor.

**Conclusion:**

Serum CST1 has the potential to function as a diagnostic indicator for distinguishing early-stage esophageal squamous cell carcinoma (ESCC) from individuals with benign esophageal lesions and healthy individuals. Additionally, it could serve as a prognostic predictor and therapeutic efficacy indicator for patients with ESCC.

## Introduction

Esophageal cancer (EC) ranks seventh among the most prevalent cancers and sixth among the leading causes of mortality worldwide (1). Esophageal squamous cell carcinoma (ESCC) represents the most frequently observed histological subtype of EC globally. In regions recognized as high-risk areas, commonly referred to as the EC belt, ESCC accounts for over 90% of cases (2). The prognosis for esophageal cancer is generally unfavorable, primarily due to late-stage diagnoses that limit the effectiveness of available treatments and increase the likelihood of recurrence or metastasis (3). Serological biomarkers play a pivotal role in the early detection of ESCC patients, owing to their accessibility, ease of detection, and widespread acceptance. However, the conventional tumor markers Carcinoembryonic Antigen.

(CEA), cytokeratin 19 fragment antigen (CYFRA21-1), and squamous cell carcinoma antigen (SCC-Ag) exhibit varying degrees of detection sensitivity or specificity, thereby constraining their clinical utility in the timely detection of esophageal squamous cell carcinoma (4, 5). Consequently, there exists a pressing demand for markers capable of early diagnosis, as well as prognostic markers to evaluate disease progression and clinical outcomes in patients with ESCC.

Next-generation transcriptome sequencing (RNA-seq) has provided a method to delineate the entire set of transcriptional aberrations in ESCC (6, 7), helping finding some tumer markers.

Mechanistic studies of cystatin functions revealed that changes in cystatin expression affect all stages of cancer progression including tumor growth, apoptosis as well as tumor invasion, metastasis and angiogenesis (8). Cystatin-SN (CST1), a member of the cystatin superfamily, acts as an inhibitor of cysteine proteases (9–11). Extensive research has consistently demonstrated that elevated levels of CST1 are linked to the diagnosis or unfavorable prognosis of various malignant tumors (12–15).

## Material and methods

### Patients and specimens

This retrospective clinical study comprised a cohort of 148 patients diagnosed with esophageal squamous cell carcinoma (ESCC) and 11 patients with other esophageal malignant tumors at Sun Yat-sen University Cancer Center (SYSUCC) between July 2015 and November 2018. Inclusion criteria for this study required a definitive pathological diagnosis of primary ESCC by two pathologists, and exclusion of patients who had undergone any form of treatment, including surgery or chemotherapy, prior to serum collection. A cohort of 68 individuals diagnosed with esophageal benign lesions were recruited from SYSUCC between July 2015 and November 2018, based on the criteria of gastroscopic identification of esophageal lesions and subsequent pathological confirmation of non-malignant nature. Control specimens were obtained from 148 healthy volunteers who did not exhibit any malignant tumors or esophageal benign lesions. All participants were asymptomatic and had no known medical conditions, and underwent standard blood tests, serum biochemistry analysis, liver function assessment, and kidney function evaluation in both the esophageal benign lesions group and the healthy control (HC) group. The second group consisted of patients with non-squamous carcinoma of the esophagus who were pathologically diagnosed at SYSUCC from July 2015 to November 2018. The same pathological diagnosis method was employed as in the ESCC group. Out of the 40 ESCC patients who underwent surgery, serum samples were obtained from 25 patients within 1-2 weeks after the procedure and from 21 patients more than 2 weeks after the surgery. The pathological stage was determined according to the staging criteria of the 7th edition of the American Joint Committee on Cancer (AJCC) (16). Prior to publication, written informed consent was obtained from the patient(s) to ensure the anonymity of their information in this article.

### Blood collection, storage, and processing

①The serum samples were collected from 148 patients with esophageal cancer, 11 patients with other esophageal malignant tumors and 68 patients with benign esophageal lesions before treatment. The serum was divided into several tubes (3-8 tubes). The volume of each tube was 300-500ul. And all of them were frozen in an ultra-low temperature (-80°C) refrigerator and thawed before use once a tube.

②The serum samples from 148 healthy volunteers were collected (the same method as above) in two days before the ELISA test, stored at 2-8°C, and used once a tube directly.

③The serum samples from 25 surgical patients were collected, stored at 1-2 weeks after surgery (the same method as①).

④The serum samples from 21 surgical patients were collected, stored over 2 weeks after surgery (the same method as①).

### RNA isolation, cDNA library preparation, RNA-seq, and analysis

Total RNA was extracted from frozen tissues using the Trizol reagent (Invitrogen, USA) according to the manufacture’s instruction. Beads with oligo (dT) were used to isolate poly(A) mRNA. First-strand cDNA was synthesized using random hexamer-primer and reverse transcriptase (Invitrogen). The second-strand cDNA was synthesized using RNase H (Invitrogen) and DNA polymerase I (New England BioLabs). Then the cDNA libraries were prepared according to Illumina’s protocols 2 and sequenced by Illumina HiSeq™ 2000. Sequence data from genomic DNA and complementary DNA were mapped to the reference human genome (hg19) using the Burrows-Wheeler Aligner and were processed using the publicly available SAMtools, Picard, and Genome Analysis Toolkit. The quantity of gene expression was calculated by the FPKM method(Fragments Per Kb per Million fragments) (17). The genes with FDR (false discovery rate) less 0.001 and change fold more than 2 fold were considered as the DEG (differentially expressed gene).

### Validation of candidate genes via the TCGA and GEPIA database

In order to validate the findings of the pilot screening conducted through RNA transcriptome sequencing, we conducted a consultation of the TCGA and GEPIA databases to identify up-regulated DEGs associated with esophageal squamous cell carcinoma (ESCC). To obtain a list of the top 25 over-expressed genes in ESCC, we visited the home page of UALCAN (https://ualcan.path.uab.edu/) and selected “ESCC” in the cancer selection column. Furthermore, we utilized the “single gene analysis” feature on the GEPIA homepage (http://gepia.cancer-pku.cn/xuanze), specifically searching for the gene “cst1”, and selected “Boxplot” in the “Expressions DIY” column. This allowed us to obtain a boxplot illustrating the relationship between CST1 and esophageal cancer (ESCA). The data source of the GEPIA database was from the UCSC Xena project and can be considered to be corrected for batch effects.

### Development of enzyme-linked immunosorbent assay for CST1

The development of an Enzyme-Linked Immunosorbent Assay (ELISA) for CST1 involved the screening of multiple antibody pairs. A double antibody sandwich ELISA was ultimately created, utilizing a rabbit polyclonal antibody (1:2000, 16025-1-AP, Proteintech) as the capture antibody and a mouse monoclonal antibody (1:6250, MAB1285-SP, R&D Systems) labeled with biotin as the detection antibody. The CST1 fusion protein (Ag9068, Proteintech) served as the standard/positive control. Additional materials used in the assay included 96-well plates (Corning), Streptavidin-HRP (Abcam), BSA (MPBio), PBS (Zhongshan Jinqiao), and TMB (tetramethylbenzidine) color reagent & stop solution (KangweiCentury).

Briefly, the rationale was a double-antibody sandwich method. The capture antibody was coated to 96-well plates (Corning) overnight and then blocked it with BSA. Subsequently, 100ul of the test samples (1:2 diluted) were added and incubated for 2h. Next, the detection antibody was added and incubated for 2h. Next, 100 ul/well of Streptavidin-HRP was added and incubated for 20 min. Finally, the substrate (TMB) solution was added, and the reaction was stopped and read at an OD of 450 nm.

### Statistical analysis

The data analysis was conducted using Statistical Package for the Social Science 26.0 (SPSS, IBM) and GraphPad Prism 9 (San Diego, USA). Nonparametric statistical tests, specifically the Mann-Whitney U-test or Kruskal-Wallis test, were employed to compare the differences in CST1 expression among two or more groups. The receiver operating characteristic curve (ROC) was constructed using SPSS, and the area under the curve (AUC), sensitivity, and specificity were employed to assess the diagnostic efficacy. ELISA Calc was utilized to generate the ELISA standard curve. The serum CST1 levels of preoperative and postoperative patients were compared using a paired t-test. Survival analysis was conducted using the Kaplan-Meier method, and the log-rank test was employed to determine survival differences between the groups. Univariate and multivariate analyses were performed using a Cox regression model. All statistical tests were two-sided, and a significance level of p < 0.05 was deemed statistically significant.

## Results

### Identification of CST1 expression in ESCC tumor tissues and adjacent tissues

**Figure 1:**
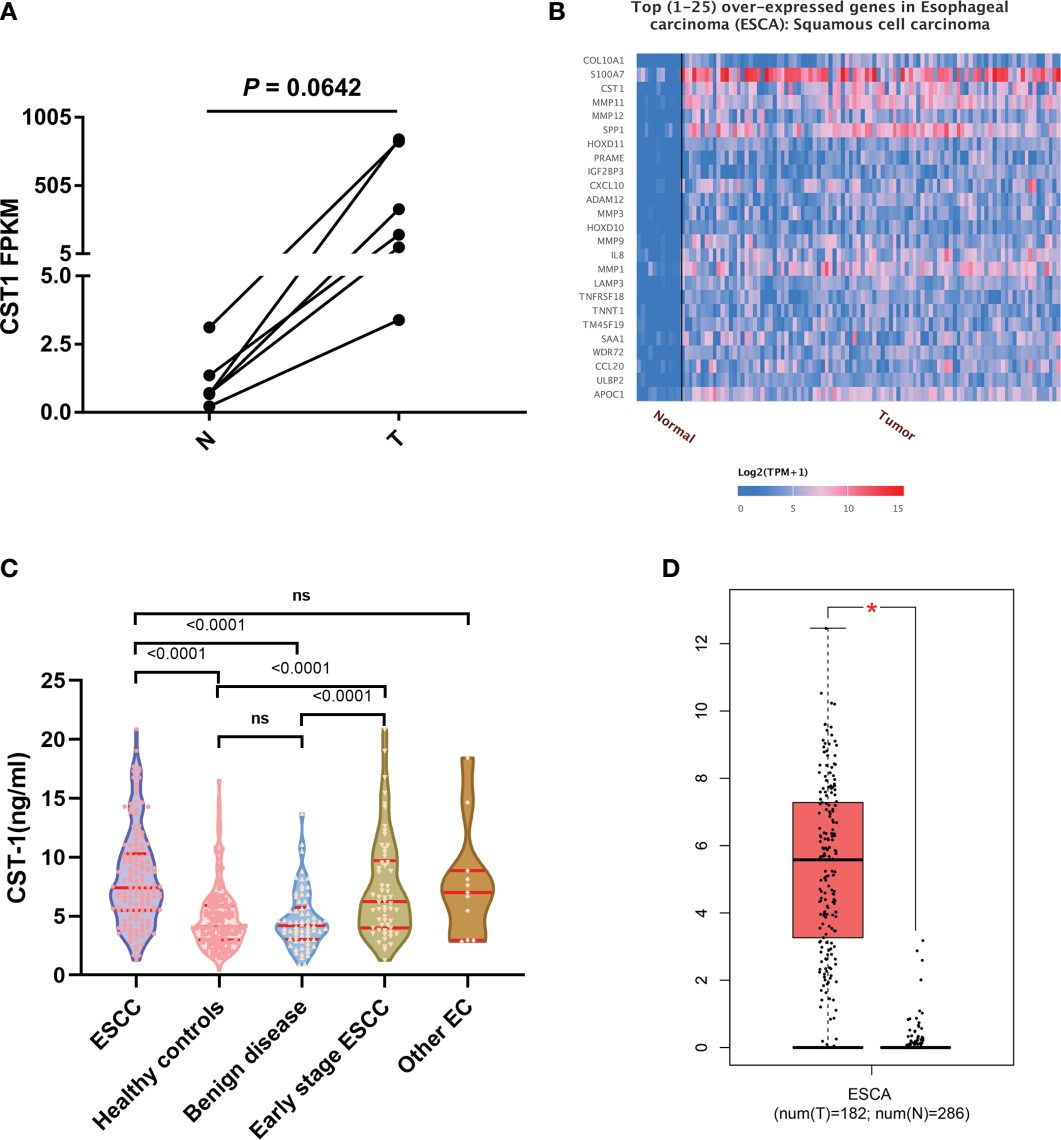
**(A)** Paired t-test of CST1 expression levels in RNA-sequencing, adjacent tissues (N) and cancer tissues (T). **(B)** the top 1-25 over expressed genes in ESCC from TCGA database. **(C)** differences of CST1 levels between different clinical groups. **(D)** the correlation between CST1gene expression and ESCA from the GEPIA database. ns, no significance; *p < 0.05.

A total of ten DEGs, including CST1, were identified through screening (18). And the expression of CST1 in ESCC tissues was consistently higher compared to adjacent tissues across all six pairs of samples (**Figure 1A**). However, the results of the paired t test did not demonstrate a statistically significant difference (p=0.0642), potentially attributed to the limited sample size.

### Verification of DEGs in ESCA from the TCGA and GEPIA database

CST1 was identified as one of the top 1-25 differentially expressed genes (DEGs) associated with esophageal squamous cell carcinoma (ESCC) based on data obtained from the TCGA database (**Figure 1B**). Additionally, a significant difference (p < 0.05) in the expression level of the CST1 gene was observed between esophageal cancer patients and individuals without the disease, as evidenced by data from the GEPIA database (**Figure 1D**).

### Serum CST1 levels in different clinical cohorts

A total of 375 participants were recruited for this study, comprising 148 patients with esophageal squamous cell carcinoma (ESCC), 68 patients with esophageal benign lesions, 148 healthy controls (107 males and 41 females), and 11 other patients with esophageal cancer (EC). The main characteristics of the 148 ESCC patients are presented in **Table 1**. Among the enrolled patients, 148 had a confirmed pathological diagnosis of ESCC, with 120 (81%) being male and 28 (19%) being female. The median age of these patients was 62 years, ranging from 44 to 83 years. The healthy controls consisted of 107 males and 41 females, and the Chi-square test showed no significant difference between the ESCC group and the HC group in gender composition (p=0.074). Notably, the serum CST1 levels exhibited variations across different clinical groups, as depicted in **Figure 1C**. The CST1 level in the ESCC group exhibited a statistically significant elevation compared to both the esophageal benign lesions group and the healthy controls (p < 0.001/< 0.001, respectively). Furthermore, the CST1 level in the early stages (stage I and II) of the ESCC group demonstrated a significant increase in comparison to the esophageal benign lesions group (p < 0.001), suggesting that CST1 possesses the ability to differentiate early esophageal cancer from benign esophageal lesions (p < 0.001). There was no statistically significant distinction observed between the ESCC group and other EC groups (such as esophageal adenocarcinoma, sarcoma, endocrine carcinoma, etc.), suggesting that CST1 may not exhibit specific elevation in ESCC compared to other types of esophageal malignant tumors. The data presented in **Table 1** indicates that there was no significant association between serum CST1 levels and variables such as gender, age, pathological differentiation degree, or alcohol consumption within the ESCC group (p > 0.05). However, a significant correlation was observed between TNM stage and serum CST1 levels (p=0.009).

**Table 1:** Clinical characteristics and serum CST1 levels of 148 ESCC patients.

	NO.	%	CST1(ng/ml)	p-value
Age in years				
<=62	78	53	7.16(1.67-20.86)	0.192
>62	70	47	8.03(1.27-19.03)	
Gender				
Male	120	81	7.57(1.27-20.86)	0.139
Female	28	19	6.25(2.79-14.29)	
TNM stage				
0III	58	39	6.25(1.27-20.86)	0.009
IIIIV	87	59	8.04(1.67-17.70)	
Unknown	3	2	5.99(5.52-9.11)	
Histological grade				
High	23	15	7.34(2.62-16.79)	0.171
Middle	99	67	7.01(1.27-17.56)	
Low	19	13	9.08(3.31-19.03)	
Unknown	7	5	10.07(1.69-20.86)	
Alcohol				
Yes	63	43	7.96(1.27-20.86)	0.140
No	68	46	6.87(2.17-17.31)	
Unknown	17	11	6.66(3.07-17.70)	

The CST1 level was expressed as the median with the range. Mann-Whitney U-test or Kruskal-Wallis test were used to compare the differences in CST1 levels between two or more than two groups.

### Diagnostic efficiency of serum CST1 for ESCC patients

**Figure 2:**
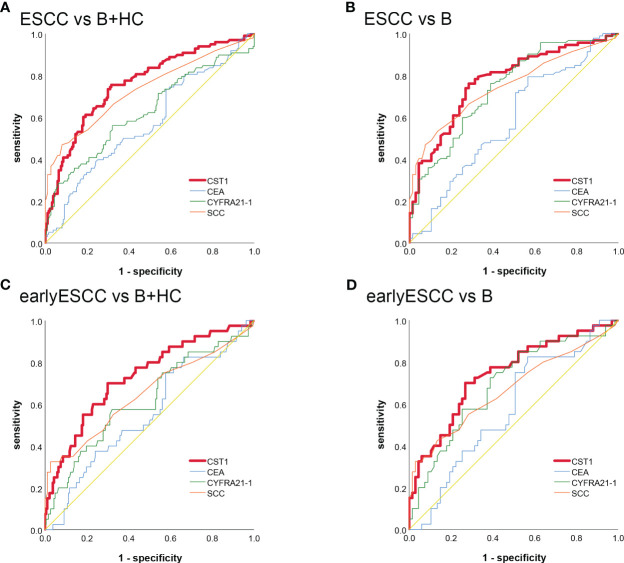
The ROC analysis of serum CST1, Cyfra21-1, CEA, and SCC indicators in distinguishing between ESCC and B+HC **(A)** ESCC and B **(B)** early-stage ESCC and B+ HC **(C)** early-stage ESCC and B **(D)**.

The area under the curve (AUC) of serum CST1 levels for distinguishing the ESCC group from the esophageal benign lesions group and healthy controls was found to be 0.782/0.775. The sensitivity and specificity were determined to be 75.68%/75.68% and 75.53%/70.83%, respectively. Additionally, the positive predictive value was calculated to be 86.15%/64.00%. When CST1 was combined with SCC, the AUC for distinguishing early ESCC from the esophageal benign lesions group and healthy controls was 0.781/0.757. The sensitivity and specificity were reported as 73.81%/69.05% and 71.64%/73.80%, respectively (**Table 2**; **Figure 2**).

**Table 2:** Results for measurement of serum CST1, CEA, CYFRA21-1, SCC or combination of CST1 and SCC in the diagnosis of ESCC.

	AUC(95%CI)	Sensitivity (%)	Specificity (%)	PPV(%)	NPV(%)
ESCC vs B and HC					
CST1	0.775(0.725, 0.824)	75.68	70.83	64.00	80.95
CEA	0.589(0.527, 0.605)	49.28	67.76	49.64	67.44
CYFRA21-1	0.675(0.615, 0.734)	59.69	67.00	53.85	72.04
SCC	0.732(0.667, 0.798)	46.94	91.98	75.41	76.79
CST1+SCC	0.819(0.766, 0.872)	69.39	79.68	64.15	83.24
ESCC vs B					
CST1	0.782(0.718, 0.846)	75.68	73.53	86.15	58.14
CEA	0.606(0.522, 0.690)	79.71	44.12	74.32	51.72
CYFRA21-1	0.753(0.681, 0.824)	77.52	61.76	79.37	59.15
SCC	0.751(0.678, 0.823)	54.08	86.57	85.48	56.31
CST1+SCC	0.840(0.781, 0.899)	61.22	91.04	90.91	61.62
Early ESCC vs B and HC					
CST1	0.696(0.617, 0.775)	63.79	70.83	37.00	87.93
CEA	0.582(0.499, 0.665)	76.79	43.46	26.22	87.74
CYFRA21-1	0.605(0.517, 0.693)	50.94	67.00	29.03	83.75
SCC	0.664(0.560, 0.767)	38.10	91.98	51.61	86.87
CST1+SCC	0.757(0.669, 0.845)	69.05	73.80	37.18	91.39
Early ESCC vs B					
CST1	0.703(0.611, 0.795)	63.79	73.53	67.27	70.42
CEA	0.592(0.491, 0.693)	85.71	43.28	55.81	78.38
CYFRA21-1	0.690(0.595, 0.785)	77.36	53.73	56.94	75.00
SCC	0.682(0.574, 0.791)	45.24	86.57	67.86	71.60
CST1+SCC	0.781(0.691, 0.872)	73.81	71.64	62.00	81.36

AUC, area under curve; PPV, positive predictive value; NPV, negative predictive value; ESCC, esophageal squamous cell carcinoma; B, benign esophageal lesions; HC, healthy controls; CST1:Cystatin-SN; CEA, carcinoembryonic antigen; CYFRA21-1, Cytokeratin 19 fragment; SCC, squamous cell carcinoma antigen.

*The diagnostic cutoff values of CST1, CEA, CYFRA21-1, SCC or combination of CST1 and SCC were 5.46ng/ml, 2.80ng/ml, 2.805ng/ml, and 1.35ng/ml, respectively.

### Serum CST1 is associated with the therapeutic effect evaluation of ESCC

**Figure 3:**
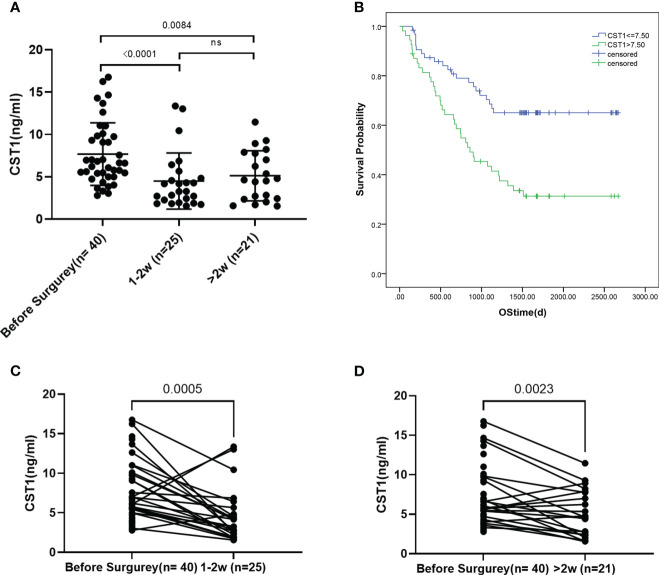
**(A)** CST1 levels of pre - and 1-2 weeks post-operation patients and over 2 weeks post-operation. **(B)** Survival analysis of ESCC patients stratified by CST1 cut-off value (7.5ng/ml). Kaplan-Meier curves show that patients with low CST1 expression had higher survival rates. **(C, D)**, Paired t-test of preoperative and postoperative CST1 levels.

A statistically significant difference was observed in the CST1 level between the preoperative period and the 1-2 weeks/> 2 weeks postoperative period (p < 0.0001/=0.0084, respectively) (**Figure 3A**). The paired t-test analysis demonstrated a significant decrease in CST1 level following the surgical procedure compared to the preoperative level (**Figures 3C, D**), suggesting that CST1 may serve as a reliable indicator of surgical effectiveness.

### Serum CST1 is associated with the prognosis of ESCC

In order to evaluate the predictive power for the prognosis of ESCC, we followed 118 ESCC participants. The characteristics of these patients were summarized in supplement table. Using the survival status of the patients as a basis, the Receiver Operating Characteristic (ROC) analysis was employed to determine the CST1 value with the maximum Area Under the Curve (AUC) as the cut-off value. Subsequently, the patients with esophageal squamous cell carcinoma (ESCC) were categorized into two groups: high serum CST1 group and low serum CST1 group. Subsequently, a comparison was made between the survival time of ESCC patients in the high and low CST1 groups. The findings revealed that ESCC patients with low CST1 levels exhibited significantly longer overall survival times compared to those with high CST1 levels (**Figure 3B**). These results suggest a positive correlation between high serum CST1 levels and poor prognosis in ESCC patients. The Cox risk regression model was employed to investigate the potential prognostic value of serum CST1 in patients with ESCC. Univariate risk regression analysis revealed significant associations between the prognosis of patients and various factors, including T stage, N stage, TNM stage, CEA, CYFRA21-1, and serum CST1 levels (**Table 3**). The multivariate risk regression analysis included four factors, namely TNM stage, serum CST1 level, serum CEA level, and serum CYFRA21-1 level. The results of the analysis revealed that serum CST1 (Hazard Ratio (HR) = 2.023; 95% confidence interval (CI) = 1.099-3.725; p = 0.024) and TNM stage (HR = 1.722; 95% CI = 1.184 – 2.505; p = 0.004) independently predict the overall survival (OS) of patients with esophageal squamous cell carcinoma (ESCC). Consequently, serum CST1 holds promise as a potential prognostic indicator for ESCC.

**Table 3:** Univariate and multivariate analysis for OS prediction of ESCC patients.

Characteristics	Univariate analysis			multivariate analysis		
	HR	95%CI	p-value	HR	95%CI	p-value
age	1.071	0.637-1.801	0.795	–	–	–
gender	0.919	0.486-1.738	0.796	–	–	–
Tumer stage	2.091	1.422-3.073	<0.001	–	–	–
Node stage	1.623	1.241-2.123	<0.001	–	–	–
TNM stage	1.964	1.409-2.739	**<0.001**	1.722	1.184-2.505	**0.004**
CST1	2.552	1.488-4.377	**0.001**	2.023	1.099-3.725	**0.024**
CEA	2.315	1.155-4.639	**0.018**	1.719	0.796-3.711	0.168
CYFRA21-1	2.779	1.439-5.370	**0.002**	1.587	0.777-3.240	0.205

Variables with p < 0.05 in the univariate analysis (TNM stage, CST1, CEA, CYFRA21-1) were entered into multivariate analysis.

The bold values in univariate analysis indicate p < 0.05, and these variables were included in the multivariate analysis. The bold values in multivariate analysis indicate p < 0.05.

In this study, the Receiver Operating Characteristic (ROC) curve was employed to determine the optimal threshold value for each factor. The Kaplan-Meier method was utilized to examine the association between serum CST1 levels and prognosis in a cohort of 118 preoperative patients diagnosed with esophageal squamous cell carcinoma (ESCC). Additionally, the log-rank test was employed to assess the disparity in survival curves between patients exhibiting high CST1 levels and those with low CST1 levels. The follow-up period for all participants extended until November 2022.

## Discussion

Esophageal cancer can be pathologically diagnosed through the use of invasive endoscopy. In China, it is suggested that people aged 40-69 years old in areas with a high incidence of esophageal cancer should be classified as a high-risk population for screening, and patients with Barrett’s esophagus or low-grade intraepithelial neoplasia should be reexamined at least every 3 years (19). The Japanese Guidelines recommended to undergo endoscopic screening every 2 to 3 years (20), as annual screening has not been endorsed by any country thus far (21). The prognosis of esophageal cancer heavily relies on the clinical stage at the time of diagnosis (22), leading to a low rate of early esophageal cancer detection and unfavorable outcomes. Consequently, the identification of serum tumor markers plays a crucial role in achieving early diagnosis of esophageal cancer.

Numerous researchers have conducted investigations on tumor transcriptomics by accessing online databases to acquire therapeutic targets and biomarkers for tumors (23). In our previous study, we conducted RNA transcriptome sequencing and successfully identified 10 genes exhibiting differential expression. However, during the validation process, only three genes demonstrated satisfactory specificity. Notably, CST1 did not exhibit a high level of specificity for ESCC. We suspect that the lack of repeat sequencing and the small sample size are possible reasons. However, after conducting a thorough search of online databases and reviewing multiple studies, it was determined that CST1 may serve as a reliable indicator for early diagnosis and prognosis of ESCC (13, 24–26). We have tried several commercial ELISA kits, performing not as expected. It may be due to the low concentration of antibody coated with the kit, or the antibody titer decreased over time. In order to address these potential concerns regarding the reliability of the ELISA results, we opted to verify them through the utilization of a self-built ELISA. Ultimately, our validation process confirmed that CST1 is indeed an exceptional marker for ESCC.

Even CST1 did not show particularly high specificity+sensitivity in **Table 2**, there are advantages compared with CEA/CYFRA21-1/SCC-Ag. Since there are no better markers for esophageal cancer up to now, CST1 is worth considered as a diagnostic marker relatively. We also look forward to the emergence of more valuable diagnostic markers for ESCC.

This study has demonstrated a significant decrease in the postoperative level of CST1 compared to the preoperative level, thereby providing further support for the validity of this diagnostic marker. Consequently, it is reasonable to consider CST1 as a potential therapeutic target. CST1 encodes Cystatin SN, a type 2 cystatin that belongs to a class of protease inhibitors commonly present in human cells and tissues (27, 28). Its involvement in various cellular processes, including cell cycle regulation, cellular senescence, tumorigenesis, and metastasis (29), suggests its significance in determining cell fate and disease progression. This inconsistency could potentially be attributed to limitations such as the small sample size or inadequate follow-up duration, necessitating further research to comprehensively investigate the relevant mechanism and draw conclusive results.

The limitations of this study encompass a limited sample size, inadequate duration of prognostic follow-up, and reliance on a single source for the sample.

## Conclusion

Serum CST1 exhibits potential utility as a diagnostic and differential diagnostic marker for ESCC. Additionally, it holds promise as an indicator of postoperative efficacy in ESCC patients. Notably, an elevated serum CST1 level is significantly correlated with unfavorable prognosis in ESCC patients, thereby suggesting its potential as a prognostic indicator for ESCC.

## Data availability statement

The data presented in the study are deposited in the RDD repository (rdd.sysu.edu.cn), accession number RDDYJ159647.

## Ethics statement

The studies involving humans were approved by the Ethics Committee of Sun Yat-sen University Cancer Center, Guangzhou, China. The studies were conducted in accordance with the local legislation and institutional requirements. The participants provided their written informed consent to participate in this study.

## Author contributions

YP: Project administration, Writing – review & editing, Conceptualization, Formal analysis. SL: Conceptualization, Writing – original draft, Writing – review & editing. XR: Validation, Visualization, Writing – review & editing. LW: Conceptualization, Writing – original draft, Writing – review & editing. YS: Formal analysis, Writing – review & editing. ZW: Visualization, Writing – review & editing. YL: Project administration, Writing – review & editing.
