# Supplementary material for: Validation of serum cystatin SN detection for diagnosis and poor prognosis of esophageal squamous cell carcinoma
Source: Front Oncol. 2024 Feb 13;14:1337707. doi: 10.3389/fonc.2024.1337707 (PMC10898351; doi:10.3389/fonc.2024.1337707)
Supplement: Supplementary file 1 [file Table_1.docx]

| **Supplementary Table: Clinical and laboratory characteristics of 118 patients associated with overall survival (OS)** | | | |
| --- | --- | --- | --- |
| characteristics | No. | Median OS (IQR) | *p* value |
| Gender |  |  |  |
| Male | 93 | 1095（493-1676） | 0.796 |
| Famale | 25 | 1280（656-1668） |  |
| Age |  |  |  |
| ≤62.1 | 61 | 1123（559-1677） | 0.794 |
| ＞62.1 | 57 | 1280（493-1673） |  |
| Tumer stage |  |  |  |
| T1 | 20 | 1686（1381-2445） | 0.001 |
| T2 | 12 | 1476（988-1687） |  |
| T3 | 75 | 974（484-1560） |  |
| T4 | 11 | 278（169-915） |  |
| Node stage |  |  |  |
| N0 | 50 | 1521（844-1824） | 0.002 |
| N1 | 39 | 1096（514-1679） |  |
| N2 | 20 | 718（336-1489） |  |
| N3 | 9 | 278（196-829） |  |
| TNM stage |  |  |  |
| Ⅰ | 17 | 1824（1536-2587） | <0.001 |
| Ⅱ | 37 | 1219（633-1543） |  |
| Ⅲ | 51 | 1061（462-1679） |  |
| Ⅳ | 13 | 382（171-902） |  |
| CST1 |  |  |  |
| ≤7.50 | 64 | 1490（645-1710） | <0.001 |
| ＞7.50 | 54 | 844（415-1543） |  |
| CEA |  |  |  |
| ≤5.24 | 99 | 1460（523-1694） | 0.015 |
| ＞5.24 | 12 | 915（640-1259） |  |
| CYFRA21-1 |  |  |  |
| ≤5.865 | 91 | 1468（586-1696） | 0.001 |
| ＞5.865 | 14 | 854（430-1321） |  |
| SCC |  |  |  |
| ≤1.3 | 41 | 1484（659-2069） | 0.361 |
| ＞1.3 | 40 | 984（497-1613） |  |
| Survival was analyzed using Kaplan–Meier method and compared with LogRank test; | | | |
| IQR: interquartile range | | | |
